# From cultivar mixtures to allelic mixtures: opposite effects of allelic richness between genotypes and genotype richness in wheat

**DOI:** 10.1111/nph.17915

**Published:** 2022-01-26

**Authors:** Germain Montazeaud, Timothée Flutre, Elsa Ballini, Jean‐Benoit Morel, Jacques David, Johanna Girodolle, Aline Rocher, Aurélie Ducasse, Cyrille Violle, Florian Fort, Hélène Fréville

**Affiliations:** ^1^ AGAP Université Montpellier CIRAD INRAE Institut Agro 34090 Montpellier France; ^2^ CEFE Université Montpellier Institut Agro CNRS EPHE IRD Université Valéry 34293 Montpellier France; ^3^ Department of Ecology and Evolution University of Lausanne 1015 Lausanne Switzerland; ^4^ Université Paris‐Saclay INRAE CNRS AgroParisTech GQE ‐ Le Moulon 91190 Gif‐sur‐Yvette France; ^5^ BGPI Université Montpellier CIRAD INRAE Institut Agro 34398 Montpellier France; ^6^ CEFE Université Montpellier CNRS EPHE IRD Université Paul Valéry 34293 Montpellier France

**Keywords:** allelic richness, crop, diversity, genome‐wide analysis, plant–plant interactions, *Septoria tritici* blotch, varietal mixtures

## Abstract

- Agroecosystem diversification through increased crop genetic diversity could provide multiple services such as improved disease control or increased productivity. However, we still poorly understand how genetic diversity affects agronomic performance.
- We grew 179 inbred lines of durum wheat in pure stands and in 202 binary mixtures in field conditions. We then tested the effect of allelic richness between genotypes and genotype richness on grain yield and *Septoria tritici* blotch disease. Allelic richness was tested at 19K single nucleotide polymorphisms distributed along the durum wheat genome. Both genotype richness and allelic richness could be equal to 1 or 2.
- Mixtures were overall more productive and less diseased than their pure stand components. Yet, we identified one locus at which allelic richness between genotypes was associated with increased disease severity and decreased grain yield. The effect of allelic richness at this locus was stronger than the effect of genotype richness on grain yield (−7.6% vs +5.7%).
- Our results suggest that positive effects of crop diversity can be reversed by unfavourable allelic associations. This highlights the need to integrate genomic data into crop diversification strategies. More generally, investigating plant–plant interactions at the genomic level is promising to better understand biodiversity–ecosystem functioning relationships.

## Introduction

Plant ecological research has long shown positive effects of biodiversity on ecosystem functioning (Loreau *et al*., 2001; Tilman *et al*., 2001; Hooper *et al*., 2005). Accordingly, more diverse agroecosystems could provide a range of services such as improved disease control or increased productivity, while reducing the use of external inputs (Jackson *et al*., 2007; Litrico & Violle, 2015). Growing mixtures of varieties instead of monogenotypic crop stands could promote such diversity effects at the intraspecific level (Barot *et al*., 2017). Most meta‐analyses indeed show that varietal mixtures have a slight yield advantage over monovarietal stands (+2% to +5%). Yet, they also highlight that mixing effects can be highly variable and even negative (Smithson & Lenné, 1996; Kiær *et al*., 2009; Reiss & Drinkwater, 2018). Understanding such variability is necessary to design optimal mixtures in which positive interactions are promoted and negative interactions are prevented (Litrico & Violle, 2015).

So far, mixing effects have mostly been studied with phenotypic approaches that aim at testing whether phenotypic diversity in a given trait can drive positive interactions through ecological mechanisms such as niche complementarity or facilitation (Loreau & Hector, 2001). For instance, diversity in plant height (Essah & Stoskopf, 2002), root traits (Montazeaud *et al*., 2018), and phenological traits (Yu *et al*., 2015) have been investigated as potential ways to promote spatial or temporal resource partitioning between crop species or genotypes. Even if some correlations between phenotypic traits and mixing effects have been reported, these approaches have not yet allowed the obtainment of general and robust assembly rules for varietal mixtures (Borg *et al*., 2018), as expected from theory (Litrico & Violle, 2015; Barot *et al*., 2017). Moreover, they are intrinsically limited by the fact that we do not know all the traits involved in plant–plant interactions. Complementary approaches have been developed from the statistical methods used in hybrid crop breeding (Gizlice *et al*., 1989; Forst *et al*., 2019). These approaches aim at estimating the mixing ability of a genotype by measuring its performance over many mixtures. Based on large mixing designs, one can identify genotypes with good mixing abilities without any prior information or hypothesis regarding their phenotype (Knott & Mundt, 1990). Even though these approaches can be very helpful for the choice of mixture components, their results are only applicable to the set of observed genotypes (although kinship‐based predictions for unobserved genotypes are theoretically possible, see Forst *et al*., 2019). Moreover, additional experiments and analyses are required if one is interested in understanding the ecological mechanisms underlying mixing ability.

One promising way to overcome the limitations of existing approaches might be to scale down to the genomic level and look for the most favourable allelic combinations. There is indeed evidence that allelic differences at specific genes between mixture components can promote positive mixing effects. For example, the well known effect of disease reduction often observed in varietal mixtures is classically attributed to allelic differences at major resistance genes between mixture components (Mundt *et al*., 1995; Gigot *et al*., 2013). The recent development of modern genomic tools has opened up new opportunities to investigate how allelic combinations underly plant–plant interactions (Subrahmaniam *et al*., 2018). For example, allelic composition at specific DNA regions associated with plant–soil interactions (Wuest & Niklaus, 2018) and flowering time (Turner *et al*., 2020) have been shown to drive stand‐level productivity in the model plant *Arabidopsis thaliana*. Although very promising in the context of varietal mixtures, such genomic approaches have not been applied in crops so far.

In this study, we used a field experiment to investigate mixing effects at the genomic level in durum wheat (*Triticum turgidum* ssp. *durum*). Durum wheat is a major staple crop that is processed into various food products, mainly pasta in European and North American countries, and couscous and bread in North African and Middle Eastern countries. As with many self‐fertilised cereals, most durum wheat varieties are inbred lines, that is each variety is fully homozygous. Here, we used 179 inbred lines derived from a population with a broad genetic basis (David *et al*., 2014), which we grew in pure stands and in 202 binary mixtures designed at random. We quantified mixing effects on two agronomic variables, grain yield and disease severity for *Septoria tritici* blotch (STB). *Septoria tritici* blotch is caused by the fungus *Zymoseptoria tritici* and it is one of the most devastating foliar disease in wheat (Fones & Gurr, 2015). Varietal mixtures have already proved efficient to reduce the severity of this disease in the field (Gigot *et al*., 2013; Kristoffersen *et al*., 2020). Then, we tested the effect of allelic richness between genotypes and genotype richness on both grain yield and STB severity. The effect of allelic richness was tested genomewide using 19K bi‐allelic single nucleotide polymorphisms (SNPs) distributed along the durum wheat genome (Rimbert *et al*., 2018), with the aim of identifying major effect loci at which allelic diversity could be associated with mixture performance. Allelic richness was defined as the number of alleles at a given SNP in the plot. As genotypes were fully homozygous and mixtures were made of two genotypes, allelic richness could be equal to 1 or 2: 1 in pure stands and in mixtures in which both components shared the same allele, and 2 in mixtures in which the components had different alleles. Genotype richness was defined as the number of inbred lines in the plot and equalled either 1 or 2: 1 in pure stands and 2 in mixtures.

## Materials and Methods

### Experimental design

**Fig. 1:**
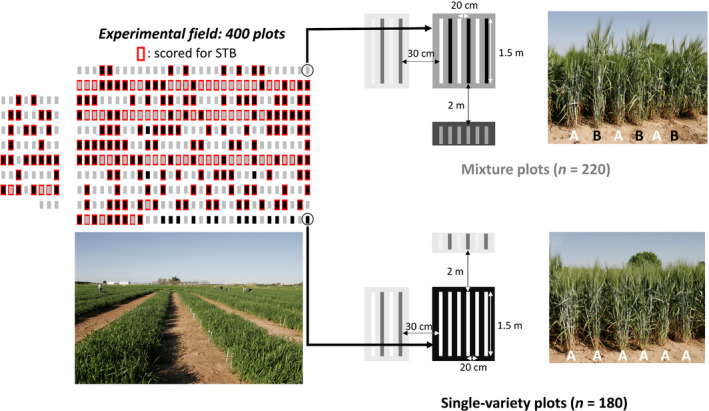
Overview of the experimental design with 400 field plots of durum wheat (*Triticum turgidum* ssp. *durum*) grown as single‐variety plots or binary mixtures. The whole design is represented on the left, with single‐variety (pure stand) plots in black, and mixture plots in grey. As shown in the figure, the design had to be split in two parts separated by *c*. 10 m to allow the irrigation system wheel to pass through. Pure stands and mixture plots were arranged randomly in the field. Plots scored for *Septoria tritici* blotch disease (STB) are framed in red. In total, 381 plots (179 pure stands and 202 mixtures) were included in the analyses after removing plots with incomplete data due to sowing or sampling problems.

We set up a field experiment at Mauguio, southern France (INRAE – UE DIASCOPE – 43°36′N, 3°59′E) on 21 November 2017. We used 179 durum wheat inbred lines from the highly diversified Evolutionary Prebreeding population (EPO) developed at INRAE Montpellier, France (David *et al*., 2014). We grew the 179 lines in pure stands and we randomly selected 202 pairwise combinations for mixture plots. We excluded pairs that had having more than a 3‐wk difference in heading date, assuming that larger time lag would not be acceptable in real cultivation conditions. Plots were not replicated because there was no requirement to test for the effects of allelic richness and genotype richness. Indeed, we rather maximised the number of replicates for both levels of genotype richness with 179 monocultures (genotype richness = 1) and 202 mixtures (genotype richness = 2). Similarly, we filtered SNPs to only keep markers with sufficient replicates for both levels of allelic richness (1 and 2, see the ‘Genotyping data’ section). Not replicating plots allowed us to increase the number of genotypes and genotype combinations in mixtures, therefore limiting potential dependency of our results on the effect of specific genotypes or genotype combinations. Pure stands and mixture plots were randomly arranged in a grid of 11 × 41 plots (Fig. 1). Each plot consisted of six 1.5 m long rows with 20 cm between rows and 2–3 cm between plants of the same row. Sixty seeds were sown on each row, resulting in a planting density of 240 plants m^−2^. The interplot distance was 30 cm in the horizontal direction and 2 m in the vertical direction. In mixtures, genotypes were grown in alternate rows, (Fig. 1). Such spatial arrangement allowed us to individualise measurements for each mixture component. Biotic damage was mitigated by applying pesticides after symptom measurements (see later). Fertilisers were used to prevent resource limitations. Detailed information on plant growth conditions, agronomic management and monthly meteorological data can be found in Supporting Information Methods S1 and Table S7 in Methods S1. The initial design comprised 400 plots with 180 monocultures and 220 mixtures, but 19 plots had to be removed from the dataset due to sowing or sampling problems.

### Productivity measurements

Productivity was quantified by measuring grain yield (g m^−2^), spike density (nb spikes m^−2^), and 1000 kernel weight (g). At maturity, we collected aboveground biomass on the four central rows of each plot on 70 cm length, leaving 40 cm on each side to avoid edge effects. For each pure stand plot, we collected two samples by pooling rows 2 and 3, and rows 4 and 5. In mixture plots, we collected two samples per genotype by separating rows 2 and 4 for the first component genotype, and rows 3 and 5 for the second component genotype. Samples were then stored in a drier for 48 h. Spikes were clipped out and counted to determined spike density. They were then threshed, and grains were weighed to determine grain yield. A subset of 250 grains was counted and weighed, and this weight was multiplied by four to obtain the 1000 kernel weight.

### Symptom measurements

We measured the impact of STB at the end of the tillering–early stem elongation stage (*c*. GS30) on 23 March 2018. By this time, no fungicides had been applied on the experiment. STB was present in almost all plots, and no other diseases were detected. We used pycnidia observations to identify STB. We scored disease severity at the plot level, which integrates the vertical and horizontal spread of the disease (Seem, 1984). The vertical spread corresponds to the infection of different leaf layers, whereas the horizontal spread corresponds to the infection of different plants. *Septoria tritici* blotch severity was assessed visually using the following scoring system: 0, no symptoms; 0.5, 5%–50% individuals with symptoms only on the low leaf layers; 1, 50%–75% individuals with symptoms only on the low leaf layers; 1.5, 75%–100% individuals with symptoms only on the low leaf layers; 2, 75%–100% individuals with symptoms on the low leaf layers and 5%–50% individuals with symptoms on the high leaf layers; 2.5, 75%–100% individuals with symptoms on the low leaf layers and 50%–75% individuals with symptoms on the high leaf layers; 3, 75%–100% individuals with symptoms on all leaf layers.

In mixture plots, the two genotypes were scored separately, and their scores were then averaged. The experiment was subdivided in three areas that were respectively scored by two interns and one experienced technician. A subset of plots was scored by all operators to compare their notations. Because intern scores were poorly correlated with the technician scores, we only retained the plots scored by the experienced technician in the analysis. We ended up with 226 plots with high‐confidence STB severity scores (166 pure stands and 60 mixtures; Fig. 1). Because almost all plots were infected by STB at this stage, we decided to apply two fungicide treatments on 20 April and 18 May to protect the experiment. We used Priori®Xtra at 1 l ha^−1^.

### Relative yield total computation

We compared the performance of the mixtures to the performance of their pure stand components using the relative yield total (RYT) index (de Wit & van den Bergh, 1965). We computed RYT for both grain yield and STB severity using the following formula:

$${\text{RYT}}_{\mathrm{ij}}=\frac{Y_{i_{\text{mixt}}}/Y_{i_{\text{monoc}}}+Y_{j_{\text{mixt}}}/Y_{j_{\text{monoc}}}}{2},$$

where: ${\mathrm{RYT}}_{\mathrm{ij}}$, relative yield total of the mixture containing genotypes *i* and *j*, $Y_{i_{\mathrm{mixt}}}$ and $Y_{j_{\mathrm{mixt}}}$, the grain yield or STB severity of genotypes *i* and *j* in mixture; $Y_{i_{\mathrm{monoc}}}$ and $Y_{j_{\mathrm{monoc}}}$, the grain yield or STB severity of genotypes *i* and *j* in pure stands. Under no mixing effects, RYT equals 1. Relative yield total > 1 means that the mixture produced more grains (grain yield) or was sicker (STB severity) than the average of the two pure stand components, whereas RYT < 1 means the opposite. Due to missing data for some plots (see the ‘Experimental design’ section), we were only able to calculate RYT for 197 (grain yield) and 46 (STB severity) mixtures, respectively.

### Genotyping data

The 179 inbred lines were genotyped with the TaBW280K high‐throughput genotyping array (Rimbert *et al*., 2018) which provided 280K SNPs. Durum wheat is allotetraploid, meaning that it combines two independent diploid genomes composed of seven chromosome pairs each (2*n* = 4*X* = 28). Nonpolymorphic SNPs and SNPs with > 5% missing values across individuals were discarded, leaving us with 117 888 SNPs. As SNPs were bi‐allelic and genotypes were inbred lines, two genotypes were observed at each locus: ‘AA’ or ‘BB’, ‘A’ and ‘B’ being the two SNP alleles.

We used this set of SNPs to compute genetic similarity between inbred lines. Indeed, EPO lines were derived from a composite‐cross population with recurrent gene flows between individuals, and were therefore genetically related to each other (David *et al*., 2014; Methods S1). To account for this nonindependence between observations in statistical analyses, we included a ‘genotype’ or a ‘genotypic pair’ random effect in our mixed models, depending on whether the response variable was measured at the individual or at the plot level, respectively. These random effects were structured according to pairwise genetic similarity matrices either computed between genotypes or between genotypic pairs. For individual‐level observations, we used the classical VanRaden additive genetic relatedness matrix (VanRaden, 2008), ***K***, which we computed with the function *estimGenRel()* from the rutilstimflutre package (Flutre & Brault, 2019). For plot‐level observations, we defined the matrix ***K_p_***, where $K_{p}$[*i*, *j*] measures the genetic similarity between genotypic pairs *i* and *j*. $K_{p}$[*i*, *j*] was computed as the probability to draw the same allele when sampling randomly an allele in each of the two pairs, averaged over all loci (Fig. S1):

(Eqn 1) $$K_{p}\left[i,j\right]=1‐\frac{1}{4L}\sum_{l=1}^{L}\left(x_{i1l}+x_{i2l}+x_{j1l}+x_{j2l}\right)+\frac{1}{8L}\sum_{l=1}^{L}\left(x_{i1l}x_{j1l}+x_{i1l}x_{j2l}+x_{i2l}x_{j1l}+x_{i2l}x_{j2l}\right)$$

$x_{i1l}$ and $x_{i2l}$, being the SNP genotypes of respectively, line 1 and line 2 at locus *l* in the plot *i*, $x_{j1l}$ and $x_{j2l}$, the SNP genotypes of respectively, line 1 and line 2 at locus *l* in the plot *j*; *L*, the total number of SNPs (*L = *117 888). Line 1 and line 2 were identical in pure stand plots. Single nucleotide polymorphisms genotypes were encoded as the number of copies of the minor allele in the pool of 179 lines, which was either 0 or 2 as we used inbred lines.

For each SNP, we then counted the number of genotypic pairs falling into each of the five possible categories: monogenotypic & monoallelic ‘AA’, monogenotypic & monoallelic ‘BB’, bi‐genotypic & monoallelic ‘AA‐AA’, bi‐genotypic & monoallelic ‘BB‐BB’, and bi‐genotypic & bi‐allelic ‘AA‐BB’. We discarded all SNPs for which the least frequent category represented less than 5% of the total dataset (*c*. 19 genotypic pairs). We ended up with 18 868 SNPs that we used to test the effect of allelic richness on grain yield. The same filtering process resulted in 6193 SNPs for STB severity as this variable was only measured in 226 plots. Single nucleotide polymorphisms physical positions and functional annotations were retrieved from the durum wheat reference genome (Maccaferri *et al*., 2019).

### Statistical analysis

All statistical analysis were performed in R v.3.5.3 (R Core Team, 2019).

#### Spatial corrections

We corrected yield components (spike density, 1000 kernel weight, and grain yield) for spatial autocorrelation at the scale of each plot row for both pure stands and mixture plots using the P‐splines method implemented in the spats package (Rodríguez‐Álvarez *et al*., 2018; Figs S2–S4). Such corrections were not possible for STB severity as measurements were not replicated within plots. We fitted a linear mixed model with genotype identity, row identity, and column identity as random effects and a smooth bivariate surface function to model the deviation to the linear trends along rows and columns. Row identity was defined as the coordinate of the plot along the smallest dimension of the grid and therefore ranged from 1 to 11 (Figs 1, S2). As yield measurements were individualised for the four central rows of each plot, each of the 41 columns of the grid was divided into four subcolumns. Column identity was defined as the subcolumn where the yield component was measured and ranged from 1 to 164 (4 × 41) (Figs 1, S2). We used the genotype best linear unbiased predictors (BLUPs) obtained from this model as response variables for all subsequent analyses, which provided us with one value per genotype per plot.

#### Detection of single‐locus allelic richness effects on grain yield and STB severity

We tested the effect of allelic richness on grain yield using an association mapping approach. For each of the 18 868 SNPs, we used the following linear mixed model:

(Eqn 2) $$y=1\mu +a\beta_{a}+g\beta_{g}+p+\varepsilon$$

where ***y*** denotes the vector of 381 observations of grain yield, *μ* is the mean grain yield across all plots, ***a*** is the vector of allelic richness, that is the number of alleles at the tested SNP (1 vs 2) across the 381 plots, and *β_a_* is the fixed effect of allelic richness. We used genotype richness as a cofactor for all SNPs, with, ***g*** being the vector of genotype richness, that is the number of inbred lines in the plot (1 vs 2) across the 381 plots, and *β_g_* being the fixed effect of genotype richness. The model was fitted sequentially with allelic richness specified before genotype richness. This allowed us to test first for allelic richness, our main factor of interest in this study, by considering that all monoallelic plots were equivalent, that is disregarding potential differences between monoallelic pure stands and monoallelic mixtures when testing the effect of allelic richness. Our design did not allow us to test for an interaction between allelic richness and genotype richness: while mixtures could be either monoallelic or bi‐allelic, pure stands were always monoallelic. We therefore ended up with three possible combinations of allelic and genotype richness: one allele and one inbred line (pure stand plots), 1 allele and two inbred lines (monoallelic mixture plots), and two alleles and two inbred lines (bi‐allelic mixtures). As mentioned before, as the 381 genotypic pairs are not independent, we included a ‘genotypic pair’ random effect, ***p***, which corresponds to the concatenation of the identity of the two genotypes present in a pair. ***p*** was assumed to be normally distributed with a mean of 0 and a variance $\sigma_{p}^{2}$, and to be structured according to a 381 × 381 pairwise genetic similarity matrix ***K_p_***. Structuring genotypic pair random effect with ***K_p_*** allowed us to control for the confounding effect of the genetic background (Vilhjálmsson & Nordborg, 2013) and to minimise the detection of false positives (Fig. S5). Finally, ***ε*** is the vector of error terms which are assumed to be independent and identically distributed with variance $\sigma_{r}^{2}$. To account for potential grain yield differences between the two alleles at the tested SNP, we initially included an ‘allelic dosage effect’, defined as the total number of minor allelic copies in the plot at the given locus. By encoding SNP alleles 0 and 1 for the major and minor alleles respectively, we defined allelic dosage value as 0 for SNP combination 0–0, 2 for combination 0–2 or 2–0, and 4 for combination 2–2. This effect was never significant and was therefore discarded.

As the 18 868 tests were not independent due to linkage disequilibrium between SNPs, we computed the effective number of independent tests for each chromosome using the Galwey method (Galwey, 2009) implemented in the *meff()* function from the poolr package. We obtained a total of 902 independent tests. Based on this number, we then limited the detection of false positive by controlling the family‐wise error rate (FWER) at 5% with the Bonferroni correction. We checked linkage disequilibrium (Fig. S6) and we analysed gene functional annotation (Dataset S1) in the genomic regions where we found significant effects of allelic richness on grain yield. To select functional annotations, we used the positions of the closest SNPs outside the significant hit as upper and lower bounds (Fig. S6) and we only considered high‐confidence annotations (Dataset S1).

We ran the exact same locus‐by‐locus analysis for STB severity, except that we used 6193 SNPs (see the ‘Genotyping data’ section) and 226 observations (Fig. S7).

For SNPs at which we detected a significant effect of allelic richness on grain yield, we used the model defined by Eqn 2 with spike density or 1000 kernel weight as response variables to assess which yield component was primarily affected by allelic richness. Also, to check if the effect on grain yield could originate from an effect on STB severity, we first tested the overall relationship between grain yield and STB severity (Table S1). We included the fixed effect of genotype richness as well as the interaction between genotype richness and STB severity to account for differences between monocultures and mixtures. Then, we used the model defined by Eqn 2 with STB severity as the response variable and allelic richness computed at the subset of SNPs with a significant effect on grain yield. For these SNPs, we also checked whether the significant effect of allelic richness on grain yield could originate from unwitting sampling effects resulting from either (1) the genotypes observed in bi‐allelic mixtures being, by chance, the genotypes with the lowest yield and the highest STB severity among the 179 genotypes used in our experiment; (2) the genotypes observed in monoallelic mixtures being, by chance, the genotypes with the highest yield and lowest STB severity; or (3) both (1) and (2). We did not detect such sampling effects (Methods S1). Finally, to identify the traits underlying the effects of allelic richness, we tested the association between allelic variation at the significant SNPs and phenotypic variation at 20 functional traits measured in single‐variety plots. The 20 traits included seven aboveground traits, 11 root traits, and two phenological traits (see Methods S1 for details on trait measurements). We used a classical genome‐wide association study (GWAS) model with the phenotypic trait as the response variable, the allelic value at the tested SNP as a fixed effect, and the identity of the genotype as a random effect. The genotype identity random effect was structured with a 179 × 179 additive genetic relatedness matrix ***K***. As we tested multiple traits, we used the Benjamini–Hochberg *P*‐value correction to limit the detection of false positives.

#### Contribution of each mixture component to single‐locus allelic richness effects

When we detected significant allelic richness effects on grain yield or STB severity, we investigated how individual genotypes within mixture plots were affected depending on their allele and the allele of their neighbour, that is the mixture partner of the focal genotype, at the significant SNP. We used the following linear mixed model:

(Eqn 3) $$y=1\mu +x_{f}\beta_{f}+x_{n}\beta_{n}+x_{fn}\beta_{\mathrm{fn}}+Z_{f}f+Z_{n}n+\varepsilon$$

where ***y***, denotes the 404 × 1 vector of grain yield or the 120 × 1 vector of STB severity measured on each mixture component referred to as the focal genotypes; *μ*, the mean value across all mixture components; ***x*_f_**, vector of focal alleles at the tested SNP; *β*_f_, the fixed effect of the focal allele; ***x*_n_**, the vector of neighbour alleles at the tested SNP; *β*_n_, the fixed effect of the neighbour allele; ***x*_fn_**, the vector of focal‐neighbour allelic combinations at the tested SNP; *β*_fn_, the fixed effect of the interaction between focal and neighbour alleles; ***f***, the random effect of the focal genotype, which accounts for the polygenic effect of all loci other than those tested in the focal genotype; ***n***, the random effect of the neighbour genotype, which accounts for the polygenic effect of all loci other than those tested in the neighbour genotype; ***Z*_f_** and ***Z*_n_**, the incidence matrices linking observations to the correct random effects; and ***ε***, the vector of error terms assumed to be independent and identically distributed with a mean of 0 and a variance $\sigma_{r}^{2}$. Focal and neighbour genotype random effects were assumed to be normally distributed with a mean of 0 and a variance $\sigma_{f}^{2}$ and $\sigma_{n}^{2}$, respectively, and to be structured with a relatedness matrix ***K***. Note that the same matrix was used for both effects because mixture components are alternatively considered as focal and neighbour genotypes in this analysis. Results from the full model are reported in Table S2. Results in the main text are presented according to the allele carried by focal individuals (Table S3).

All mixed models were fitted with the *lmerAM()* function from the rutilstimflutre package (Flutre & Brault, 2019). We estimated marginal means ($\hat{\mu }$) for each factor levels using the *emmeans()* function from the emmeans package. We computed partial coefficients of determination $\left(R_{p}^{2}\right)$ for fixed effects using the *r2beta()* function from the r2glmm package. Post‐hoc multiple comparisons were tested with the *glht()* function from the multcomp *()* package.

## Results

**Fig. 2:**
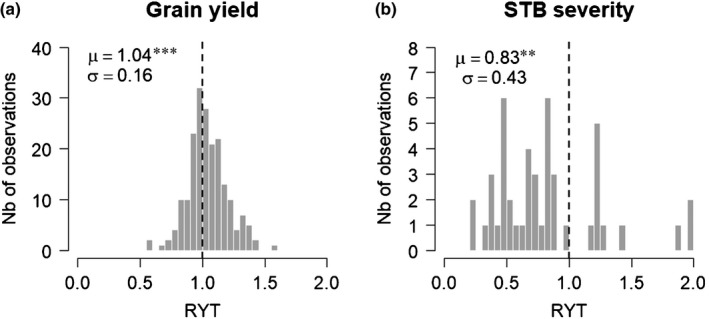
Distribution of mixing effects in field‐grown varietal mixtures of durum wheat (*Triticum turgidum* ssp. *durum*). Mixing effects are reported for grain yield (a, *n* = 197) and *Septoria tritici* blotch disease (STB) severity (b, *n* = 46). Mixing effects were quantified with the relative yield total (RYT) index. Means (*μ*) and standard deviations (σ) are reported. The star symbol indicates a mean RYT significantly different from 1 (*t*‐test, **, *P* ≤ 0.01; ***, *P* ≤ 0.001).

On average, mixtures had higher yields (+4%, *P* < 0.001) and were significantly less affected by STB (−17%, *P* = 0.0091) than expected from their pure stand components (Fig. 2). However, mixing effects were highly variables: 43% of the mixtures were less productive than expected from their pure stand components (Fig. 2a), and 24% of the mixtures were more affected by STB than expected from their pure stand components (Fig. 2b).

**Fig. 3:**
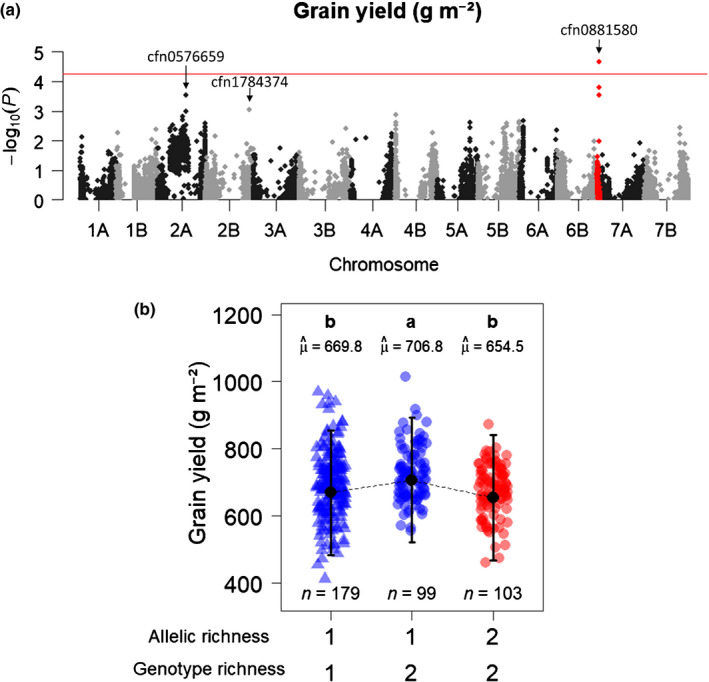
Effects of allelic richness and genotype richness on grain yield in durum wheat (*Triticum turgidum* ssp. *durum*). (a) Manhattan plot reporting *P*‐values (−log_10_ transformed) for the association tests between grain yield and allelic richness at 18 868 SNPs distributed along the durum wheat genome. The solid red line represents the family‐wise error rate (FWER) of 5% computed with the Galwey method. (b) Grain yield over the different combinations of allelic richness at cfn0881580 and genotype richness. Genotype richness is quantified as the number of genotypes in the plot, while allelic richness is quantified as the number of alleles in the plot. Point shapes: triangle, pure stand plots; circles, mixture plots. Point colours: blue, monoallelic plots; red, bi‐allelic plots. Black points and error bars represent the estimated marginal means and their 95% confidence interval. *n*, number of observations in each category, $\hat{\mu }$, marginal means. Categories with different letters are significantly different at *P* < 0.05 (Tukey adjustment). Detailed results of the statistical analyses can be found in Supporting Information Table S4.

**Fig. 4:**
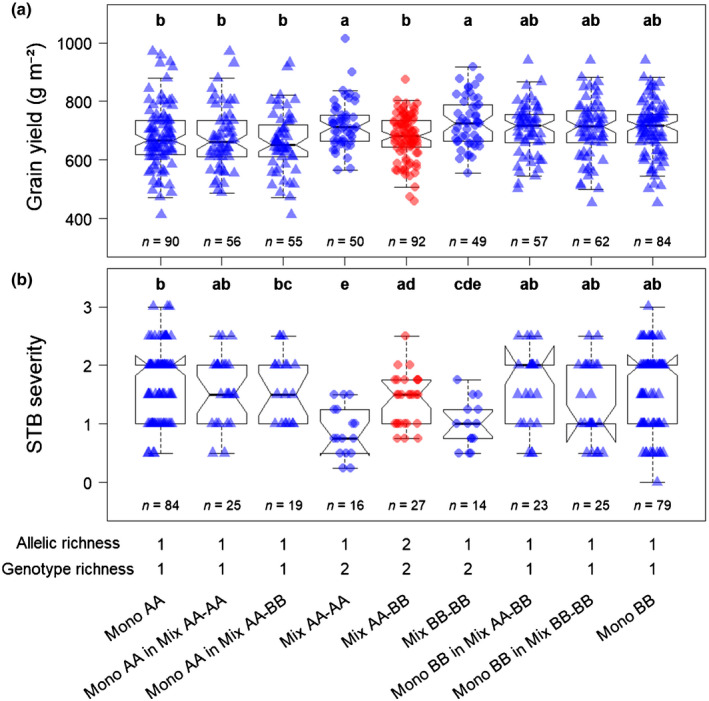
Detailed effects of allelic richness at cfn0881580 and genotype richness on grain yield (a) and *Septoria tritici* blotch disease (STB) severity (b) in durum wheat (*Triticum turgidum* ssp. *durum*). Here we compare grain yield and STB severity, both measured at the plot level, among different subsets of pure stand and mixture plots: all pure stands in which genotypes carried the ‘A’ allele at cfn0881580 (‘Mono AA’), subset of pure stands in which genotypes carried the ‘A’ allele and were used in monoallelic mixtures (‘Mono AA in Mix AA‐AA’), subset of pure stands in which genotypes carried the ‘A’ allele and were used in bi‐allelic mixtures (‘Mono AA in Mix AA‐BB’), mixtures in which both genotypes carried the ‘A’ allele (‘Mix AA‐AA’), mixtures in which one genotype carried the ‘A’ allele and the other genotype carried the ‘B’ allele (‘Mix AA‐BB’), mixtures in which both genotypes carried the ‘B’ allele (‘Mix BB‐BB’), subset of pure stands in which genotypes carried the ‘B’ allele and were used in bi‐allelic mixtures (‘Mono BB in Mix AA‐BB’), subset of pure stands in which genotypes carried the ‘B’ allele and were used in monoallelic mixtures (‘Mono BB in Mix BB‐BB’), and all pure stands in which genotypes carried the ‘B’ allele (‘Mono BB’). Point shapes: triangles, pure stand plots; circles, mixture plots. Point colours: blue, monoallelic plots; red, bi‐allelic plots. *n*, number of observations in each category. Categories with different letters are significantly different at *P* < 0.05 (Tukey adjustment).

**Fig. 5:**
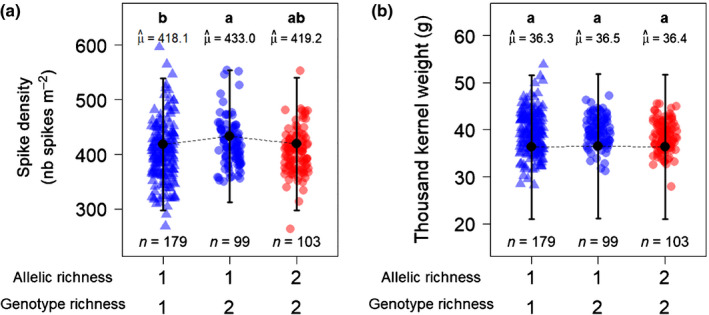
Effects of allelic richness at cfn0881580 and genotype richness on spike density (a) and 1000 kernel weight (b) in durum wheat (*Triticum turgidum* ssp. *durum*). Both spike density and 1000 kernel weight are compared over the different combinations of allelic richness at cfn0881580 and genotype richness. Genotype richness is quantified as the number of genotypes in the plot, while allelic richness is quantified as the number of alleles at in the plot. Point shapes: triangles, pure stand plots; circles, mixture plots. Point colours: blue, monoallelic plots; red, bi‐allelic plots. Black points and error bars represent the estimated marginal means and their 95% confidence interval. *n*, number of observations in each category, $\hat{\mu }$, marginal means. Categories with different letters are significantly different at *P* < 0.05 (Tukey adjustment). Detailed results of the statistical analyses can be found in Supporting Information Table S5.

At the genomic scale, we detected a single major effect locus located around SNP cfn0881580 (chr. 6B) at which allelic richness was significantly associated with grain yield (*P* = 2.1772 × 10^−5^; Fig. 3a). Plots in which genotypes had two different alleles at this locus were significantly less productive than plots in which both genotypes shared the same allele (Fig. 3b; Table S4). Moreover, the two types of monoallelic plots at cfn0881580 (AA‐AA and BB‐BB), showed similar productivities (Fig. 4a). The joint effect of genotype richness was positive: plots with two genotypes were on average more productive than plots with a single genotype when accounting for allelic richness at cfn088580 (*P* < 0.001; Fig. 3b; Table S4). This means that the yield advantage of the mixtures was not only a relative to their components grown in pure stands (Fig. 2a), but also absolute compared with all pure stands (Fig. 3b). Overall, the effect of allelic richness at cfn0881580 was opposite and stronger than the effect of genotype richness on grain yield (−7.6% ± 1.8% vs +5.7% ± 1.7%, Fig. 3b; Table S4). Moreover, both allelic richness at cfn0881580 and genotype richness primarily affected grain yield through spike density, with no effect on 1000 kernel weight (Fig. 5; Table S5).

**Fig. 6:**
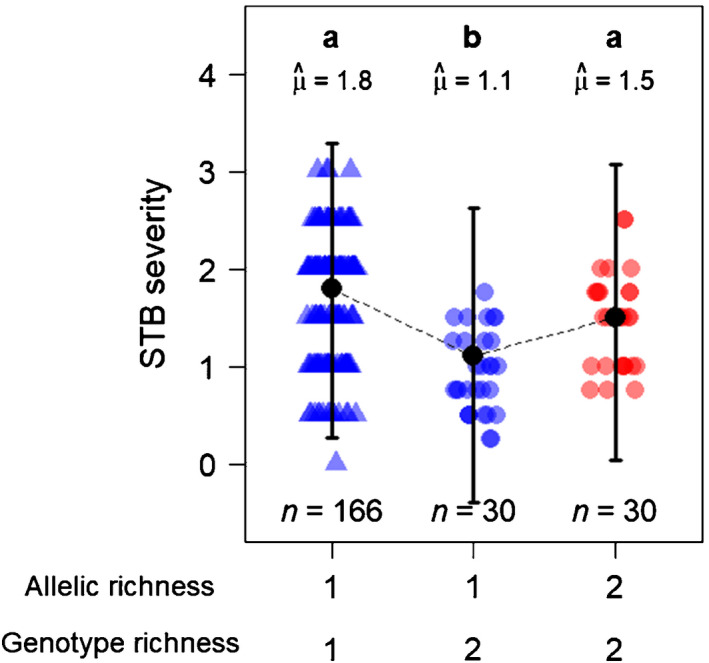
Effects of allelic richness at cfn0881580 and genotype richness on *Septoria tritici* blotch disease (STB) severity in durum wheat (*Triticum turgidum* ssp. *durum*). *Septoria tritici* blotch severity is compared over the different combinations of allelic richness at cfn0881580 and genotype richness. Genotype richness is quantified as the number of genotypes in the plot, while allelic richness is quantified as the number of alleles at in the plot. Point shapes: triangles, pure stand plots; circles, mixture plots. Point colours: blue, monoallelic plots; red, bi‐allelic plots. Black points and error bars represent the estimated marginal means and their 95% confidence interval. *n*, number of observations in each category, $\hat{\mu }$, marginal means. Categories with different letters are significantly different at *P* < 0.05 (Tukey adjustment). Detailed results of the statistical analyses can be found in Supporting Information Table S4.

The effect of STB severity on grain yield was not significant overall (Fig. S8; Table S1). Also, the genome‐wide scan did not detect any locus with significant allelic richness effect on STB severity (Fig. S7). However, when considering only cfn0881580, allelic richness was significantly associated with STB severity: plots combining two alleles at cfn0881580 were more impacted by STB than monoallelic plots (+30.7% ± 10%, *P* = 0.002; Fig. 6; Table S4). The two alternative alleles at cfn0881580 showed similar STB severities in monoallelic plots (Fig. 4b). As observed for grain yield, genotype richness had the opposite effect of allelic richness on STB severity: mixture plots were less impacted by STB than pure stands (−33.5% ± 5.5%, *P* < 0.001; Fig. 6; Table S4).

The effect of allelic richness at cfn0881580 could not be explained by intrinsic differences in yield or STB susceptibility between the genotypes used in monoallelic vs bi‐allelic plots (Fig. 4), therefore suggesting that differences between monoallelic and bi‐allelic plots resulted from an interaction between the alleles carried by the two genotypes.

**Fig. 7:**
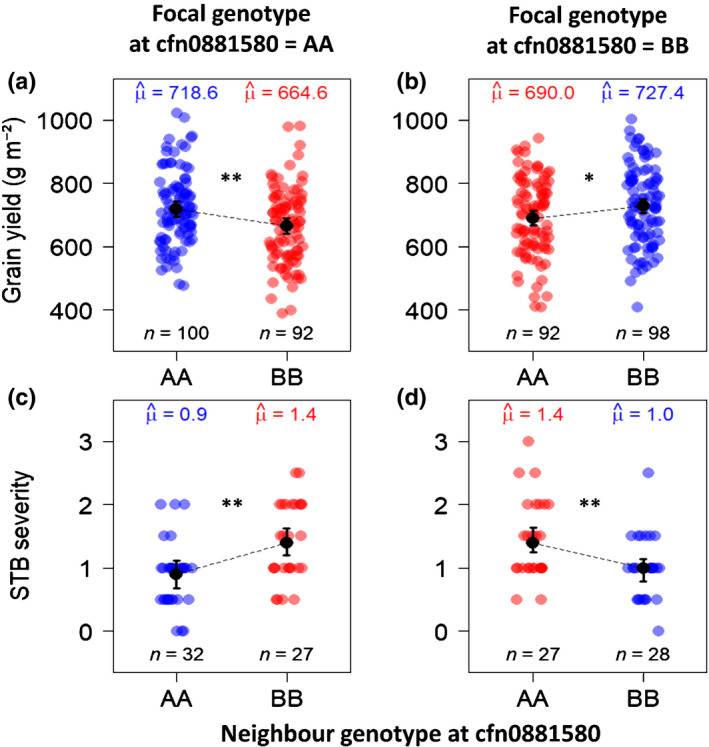
Effect of the interaction between focal allele and neighbour allele at cfn0881580 on grain yield (a, b) and *Septoria tritici* blotch disease (STB) severity (c, d) in mixture plots of durum wheat *(Triticum turgidum* ssp. *durum)*. Neighbour allele (genotype) here refers to the allele (genotype) carried by the mixture partner of the focal genotype. Point colours: blue, grain yield (a, b) or STB severity (c, d) measured on mixture components that shared the same allele as their neighbours, red, grain yield (a, b) or STB severity (c, d) measured on mixture components that carried a different allele than their neighbours. *n*, number of observations in each category, $\hat{\mu }$, marginal means. Black points and error bars represent the estimated marginal means and their 95% confidence interval. Statistical significance is highlighted with black stars (*, *P* ≤ 0.05; **, *P* ≤ 0.01). Detailed results of the statistical analyses can be found in Supporting Information Tables S2, S3.

When analysing mixture plots only, we did detect a strong interaction between the alleles carried by the two components of the mixture at cfn0881580 (Fig. 7; Tables S2, S3): genotypes were significantly less productive (Fig. 7a,b) and more diseased (Fig. 7c,d) when grown with a mixture partner carrying a different allele at cfn0881580.

To check if the results observed at cfn0881580 were specific to this locus, we also analysed the effect of allelic richness at the two most significant SNPs outside the peak on chromosome 6B, namely cfn0576659 on chromosome 2A and cfn1784374 on chromosome 2B (Figs 3, S9). As for cfn0881580, mixture plots with two different alleles at these SNPs were, on average, less productive than mixtures plots where a single allele was shared by both genotypes (*P* = 2.9087 × 10^−4^ and *P* = 9.1756 × 10^−4^, respectively; Fig. S9b,c). We found no relationship between allelic richness at cfn0576659 and STB severity (Fig. S9d). We could not check the relationship between allelic richness at cfn1784374 and STB severity because this SNP had very unbalanced allelic representation in the STB dataset.

We did not find any association between allelic variation at cfn0881580 and phenotypic variation at the 20 functional traits measured in the experiment (Table S6).

## Discussion

Ecological theories suggest that greater crop diversity can provide multiple benefits to agriculture, including greater productivity and reduced disease severity (Altieri, 1999; Jackson *et al*., 2007; Hajjar *et al*., 2008). In this study, we confirmed longstanding results showing that such benefits can be obtained by mixing different genotypes within the same field. Indeed, we report an overall benefit of genotype diversity for both grain yield and STB severity in wheat varietal mixtures grown in field conditions. However, mixing crop genotypes randomly does not seem sufficient to benefit from positive diversity effects, as shown by the considerable variability in mixing effects classically reported in previous studies on varietal mixtures (Smithson & Lenné, 1996; Kiær *et al*., 2009; Reiss & Drinkwater, 2018). We here confirmed this pattern by reporting significant variation in productivity and disease severity (both absolute and relative to monocultures). Research efforts are then still needed to define general guidelines for the design of optimised multifunctional mixtures (Litrico & Violle, 2015). So far, the vast majority of these efforts have tried to identify the most favourable combinations of traits, or the best genotypes to assemble based on their mixing abilities (Barot *et al*., 2017). However, such approaches might have overlooked lower‐scale interactions occurring at the genomic level. In this study, we show for the first time in crops that allelic richness at a single locus can have major negative effects on varietal mixture performance.

We identified one major effect locus at which allelic differences between genotypes were associated with decreased grain yield and increased STB severity. While such negative effect of allelic richness at a single locus had never been reported in crops, a similar pattern was recently documented in the model plant *Arabidopsis thaliana* (Turner *et al*., 2020), where allelic differences between mixture components led to underyielding. We observed similar negative associations between allelic diversity and grain yield at two other SNPs that were the closest from the significance level. Overall, this suggests that negative associations between allelic diversity and crop performance might not be uncommon. Such opposite effects of genotype richness and allelic richness at specific loci might explain why meta‐analyses on varietal mixtures have reported both positive and negative mixing effects on mixture productivity (Smithson & Lenné, 1996; Kiær *et al*., 2009; Reiss & Drinkwater, 2018). Importantly, our results showed that such negative effects of allelic diversity at a single locus do not preclude an overall positive effect of genetic diversity, as shown by the average increase in grain yield and decrease in STB severity associated with genotype richness. However, the yield reduction associated with allelic richness was stronger than the yield increase associated with genotype richness. This suggests that beneficial mixing effects arising from genotype diversity can be cancelled and even reversed by unfavourable allelic associations. Therefore, our findings do not question the benefits of crop diversity for agriculture, but rather provide a new perspective on the relative contributions of different levels of diversity to these benefits.

Analysing diversity effects at the allelic level can help the understanding of the ecological basis of mixing effects. For example, Turner *et al*. (2020) showed that the gene associated with community‐level biomass in *A. thaliana* was a major flowering time locus, and they could therefore attribute mixture underyielding to differences in flowering time between accessions. In our case, we did not find direct associations between allelic variation at the identified locus and any of the phenotypic traits measured in the experiment. However, the traits were measured in monocultures only and the effect of allelic richness was primarily detected in mixtures. Then, phenotypic plasticity between monocultures and mixtures could explain why we did not find any association with this locus (Dahlin *et al*., 2020). Our results however suggested that the yield reduction observed in bi‐allelic mixtures was a direct consequence of higher STB severity. The early timing of STB infection combined with the fact that allelic differences mostly affected spike density together supported this hypothesis. Indeed, STB infections occurring around tillering are known to reduce spike density, either through a decrease in tiller production, or through a reduction in tiller survival (Leitch & Jenkins, 1995; Simón *et al*., 2002; Castro & Simón, 2016). Interestingly, while multiple studies previously reported significant mixing effects on both yield and disease severity, it has been challenging to relate these two effects (Mille *et al*., 2006; Gigot *et al*., 2013; Kristoffersen *et al*., 2020). Similarly, the overall effect of STB severity on grain yield was not significant in this study, highlighting the relevance of the genome‐wide approach to better understand the connection between yield and disease. More generally, analysing diversity effects at the genomic scale could help us to better understand the interrelationships between multiple services provided by crop diversity.

In agreement with the plant pathology literature (Mille *et al*., 2006; Gigot *et al*., 2013; Kristoffersen *et al*., 2020), we detected an overall reduction of STB severity in mixtures compared with pure stands. In previous studies (Mundt *et al*., 1995; Zeller *et al*., 2012; Gigot *et al*., 2013), this effect was mainly attributed to allelic diversity at major resistance genes among mixture components. Yet, at the genomic scale, we did not find any locus at which allelic differences between individuals significantly reduced STB severity. As the durum wheat population used in this study is very likely to contain major resistance genes for STB (see QTLs reported in Ballini *et al*., 2020), this result could be explained either by the absence of molecular markers in close linkage disequilibrium with such genes, or by the fact that STB reduction in mixtures was otherwise caused by diversity in quantitative traits (i.e. traits encoded by many loci with small effects). For example, diversity at quantitative resistance loci has already been shown to reduce STB epidemics in varietal mixtures (Cowger & Mundt, 2002).

Both alleles at cfn0881580 were found to provide lower STB severity and higher yields when grown in the vicinity of an identical allelic copy. This pattern could result either from a positive interaction between identical alleles or from a negative interaction between nonidentical alleles. In the former case, genotypes carrying the same allele could have increased the productivity of their neighbours by stimulating their immunity. Such an effect has already been reported in *Artemisia tridentata* for which plant defences have been shown to be triggered by volatile signals produced by related individuals (Karban & Shiojiri, 2009; Karban *et al*., 2013). In the latter case, plants could have produced allelopathic compounds that were toxic only to neighbours sharing a different allele. Interestingly, among the functional annotations documented in the genomic region of cfn0881580 (Dataset S1), we found an enzyme involved in the synthesis of 4‐hydroxybenzoic acid (4‐HBA), a common allelopathic compound in cereals (Guenzi & McCalla, 1966; Chou & Patrick, 1976). Whatever the nature of interactions between alleles, our results were consistent with the greenbeard effect described in evolutionary biology, in which a single gene (or cluster of tightly genes) could favour its own transmission by making individuals either more altruistic towards other individuals sharing the same gene copy or more harmful towards individuals bearing a different copy (Hamilton, 1964). In any case, further experimental work will be required to replicate this result in a different year/location, and then to decipher the fine‐scale mechanisms responsible for such negative allelic interactions.

By disentangling genetically driven positive and negative interactions among individuals, genomic approaches offer interesting perspectives for leveraging crop diversity in agroecological practices. Notably, varietal mixture composition could be optimised by using marker‐assisted assembly rules. Such genomic approaches of plant diversity could also allow us to decipher the genetic and molecular basis of plant–plant interactions in natural communities. Overall, our study demonstrates that investigating plant–plant interactions at the genomic level may therefore be relevant for tackling both applied issues in the agricultural context, and forefront research questions in plant biology.

## Author contributions

GM, CV, FF and HF designed the experiment. GM, AR, FF and HF measured productivity. EB, J‐BM and AD measured STB severity. GM conducted the analyses with significant support from TF. JG provided bioinformatic support to investigate gene annotations. Inputs from J‐BM, JD, CV and HF helped to interpret the results. GM led the writing of the manuscript. All authors contributed critically to the drafts and gave final approval for publication.

## Supporting information


**Dataset S1** Gene functional annotations in the genomic region surrounding single nucleotide polymorphisms (SNP) cfn0881580.

**Fig. S1** Pairwise genetic similarity between genotypic pairs.
**Fig. S2** Trends of spatial autocorrelation for grain yield.
**Fig. S3** Trends of spatial autocorrelation for spike density.
**Fig. S4** Trends of spatial autocorrelation for 1000 kernel weight.
**Fig. S5** *P*‐value distribution for the association tests between allelic richness and grain yield.
**Fig. S6** Local linkage disequilibrium in the region surrounding single nucleotide polymorphism (SNP) cfn0881580.
**Fig. S7** *P*‐value distribution for the association tests between allelic richness and *Septoria tritici* blotch (STB) severity.
**Fig. S8** Pairwise correlations between yield components and *Septoria tritici* blotch (STB) severity.
**Fig. S9** Effect of allelic richness at the two most significant single nucleotide polymorphisms (SNPs) after cfn0881580.
**Methods S1** Wheat population history, plant growth conditions, trait measurements, statistical analysis.
**Table S1** Mixed model results: effect of *Septoria tritici* blotch disease (STB) severity and genotype richness on grain yield.
**Table S2** Mixed model results: effect of the interaction between the focal and neighbour alleles at cfn0881580 on grain yield and *Septoria tritici* blotch disease (STB) severity.
**Table S3** Mixed model results: effect of the neighbour alleles at cfn0881580 on grain yield and *Septoria tritici* blotch disease (STB) severity of the focal genotype.
**Table S4** Mixed model results: effects of allelic richness at cfn0881580 and genotype richness on grain yield and *Septoria tritici* blotch disease (STB) severity.
**Table S5** Mixed model results: effects of allelic richness at cfn0881580 and genotype richness on spike density and 1000 kernel weight.
**Table S6** Association between single nucleotide polymorphisms (SNP) cfn0881580 and 20 functional traits measured in single‐variety plots.
**Table S7** Meteorological data.
Please note: Wiley Blackwell are not responsible for the content or functionality of any Supporting Information supplied by the authors. Any queries (other than missing material) should be directed to the *New Phytologist* Central Office.

## Data Availability

The datasets generated during this study and the code developed for statistical analyses are available in Zenodo with the identifier https://doi.org/10.5281/zenodo.5393959.
