# Supplementary material for: From cultivar mixtures to allelic mixtures: opposite effects of allelic richness between genotypes and genotype richness in wheat
Source: New Phytol. 2022 Jan 26;233(6):2573–84. doi: 10.1111/nph.17915 (PMC9306887; doi:10.1111/nph.17915)
Supplement: Supplementary file 2 — Fig. S1 Pairwise genetic similarity between genotypic pairs. Fig. S2 Trends of spatial autocorrelation for grain yield. Fig. S3 Trends of spatial autocorrelation for spike density. Fig. S4 Trends of spatial autocorrelation for 1000 kernel weight. Fig. S5 P‐value distribution for the association tests between allelic richness and grain yield. Fig. S6 Local linkage disequilibrium in the region surrounding single nucleotide polymorphism (SNP) cfn0881580. Fig. S7 P‐value distribution for the association tests between allelic richness and Septoria tritici blotch (STB) severity. Fig. S8 Pairwise correlations between yield components and Septoria tritici blotch (STB) severity. Fig. S9 Effect of allelic richness at the two most significant single nucleotide polymorphisms (SNPs) after cfn0881580. Methods S1 Wheat population history, plant growth conditions, trait measurements, statistical analysis. Table S1 Mixed model results: effect of Septoria tritici blotch disease (STB) severity and genotype richness on grain yield. Table S2 Mixed model results: effect of the interaction between the focal and neighbour alleles at cfn0881580 on grain yield and Septoria tritici blotch disease (STB) severity. Table S3 Mixed model results: effect of the neighbour alleles at cfn0881580 on grain yield and Septoria tritici blotch disease (STB) severity of the focal genotype. Table S4 Mixed model results: effects of allelic richness at cfn0881580 and genotype richness on grain yield and Septoria tritici blotch disease (STB) severity. Table S5 Mixed model results: effects of allelic richness at cfn0881580 and genotype richness on spike density and 1000 kernel weight. Table S6 Association between single nucleotide polymorphisms (SNP) cfn0881580 and 20 functional traits measured in single‐variety plots. Table S7 Meteorological data. Please note: Wiley Blackwell are not responsible for the content or functionality of any Supporting Information supplied by the authors. Any queries (other than missin [file NPH-233-2573-s002.pdf]

SUPPORTING INFORMATION FOR

From cultivar mixtures to allelic mixtures: opposite effects of allelic richness between genotypes and genotype richness in wheat

Germain Montazeaud[1,2,3,†], Timothée Flutre[4], Elsa Ballini[5], Jean-Benoit Morel[5], Jacques David[1], Johanna Girodolle[1], Aline Rocher[1], Aurélie Ducasse[5], Cyrille Violle[6], Florian Fort[2], Hélène Fréville[1]

[1] AGAP, Univ. Montpellier, CIRAD, INRAE, Institut Agro, 34090 Montpellier, France

[2] CEFE, Univ. Montpellier, Institut Agro, CNRS, EPHE, IRD, Univ Valéry, 34293 Montpellier, France

[3] Department of Ecology and Evolution, University of Lausanne, 1015 Lausanne, Switzerland

[4] Université Paris-Saclay, INRAE, CNRS, AgroParisTech, GQE - Le Moulon, 91190, Gif-sur-Yvette, France

[5] BGPI, Univ. Montpellier, CIRAD, INRAE, Institut Agro, 34398 Montpellier, France

[6] CEFE, Univ. Montpellier, CNRS, EPHE, IRD, Univ Paul Valéry, 34293 Montpellier, France

[†]Corresponding author: g.montazeaud@gmail.com

Article acceptance date: 6 December 2021

Methods S1

Methods S1 provides detailed information on several points presented in the Material and Methods of the main manuscript, including a description of the wheat population, plant growth conditions, and trait measurement methods, as well as additional details on statistical analysis.

Plant material

We used the EPO (Evolutionary Pre-breeding pOpulation) durum wheat panel developed at INRAE Montpellier, France (David *et al.*, 2014). EPO was obtained from a composite cross population in which a male sterility recessive nuclear gene was segregating. In 1997, genetic diversity from wild and primitive *Triticum turgidum* subspecies was introduced in that population. Over 12 years, seeds have been collected on both outcrossed sterile plants (20%) and self-fertilized hermaphrodite plants (80%), and then bulked to constitute the next generation. In 2009, 500 spikes were extracted and underwent five generations of selfing to obtain inbred lines. Based on genotypic data, 180 inbred lines were chosen to constitute a core collection that encompassed most of the genetic variability present in the original 500 lines. Overall, EPO was selected to capture a high proportion of the phenotypic variability of the *Triticum turgidum* subspecies. While remaining large compared to elite genotypes, this variability was reduced for a few traits such as plant height and heading date to stay within an acceptable range of phenotypic values for cultivation conditions. Such an evolutionary history resulted in a set of fixed lines that are well adapted to cultivation and harbor a broad phenotypic diversity. We used 179 out of the 180 lines in the experiment (1 line was lost over the course of the experiment).

Plant growth conditions

Plots were sown on November 21, 2017 and harvested between June 25 and 27, 2018. Soil was stony loam with about 1% organic matter and a pH of 8.7. Nitrogen fertilization was applied on February 22, 2018 and April 23, 2018 with 109 kg.ha^{-1} and 87 kg.ha^{-1} of Nexen® (46 % urea nitrogen). Broad leaf herbicides and graminicides were applied on March 23, 2018 with Pointer®UltraSX® (30 g.ha^{-1}) and Auzon®Duo (1 L.ha^{-1}). Two fungicide treatments were applied on April 20, 2018 and May 18, 2018 with Priori®Xtra (1 L.ha^{-1}). Insecticides were applied on May 18, 2018 with Karate®Xflow (0.063 L.ha^{-1}). The mean temperature over the growing season was 13.6 °C with a minimum temperature of -6 °C and a maximum temperature of 31.5°C. The cumulated precipitation between November 2017 and June 2018 was 633 mm (see Table S7 for monthly meteorological data).

Measurement of functional traits

We characterized the 179 genotypes in single-variety plots with 20 functional traits. Root traits (except root angle), early biomass per capita (Ear. bio.), tiller number per capita (Till. nb.), specific leaf area (SLA), and leaf nitrogen content (LNC) were measured at the end of the tillering stage. We sampled two soil cores (10 cm diameter and 15 cm depth) randomly in each plot avoiding external rows (1 and 6). We then counted the number of individual plants within each core before cutting aboveground biomass and washing roots to remove soil particles. For each aboveground sample, we randomly collected four foliar discs using a punch tool of 6 mm diameter. All discs and the remaining biomass were then dried separately at 60°C for 48h before being weighed to determine SLA (ratio between leaf area and leaf dry mass) and early biomass per capita (Ear. bio.). We also counted the number of tillers per capita (Till. nb.), and we estimated LNC by measuring spectral reflectance on the four foliar discs with a LabSpec® 4 equipped with bifurcated probe. Spectra were converted into LNC values using an adjusted version of the calibration detailed in Ecarnot *et al.*, 2013.

After sampling, roots were washed and stored in water at -18°C before treatment. After defrosting, we stained root samples with methyl violet. We then separated the seminal and the adventitious root systems that we scanned at 800 dpi. Using WinRHIZO pro (Version 2009; Regent Instrument, Quebec, Canada), we estimated the number of root tips, the root length distribution among diameter classes, and the total root length, surface, and volume. Roots were then dried for 48h at 60°C and weighed. Mean root diameters ($Diam_{sem}$ and $Diam_{adv}$) were computed as the mean of the median root diameters of each diameter class weighted by the root length in each class. Specific root lengths (SRL_{sem} and SRL_{adv}) were computed as the ratio between total root length and root dry mass, root tissue densities (RTD_{sem} and RTD_{adv}) as the ratio between root dry mass and root volume, root length densities (RLD_{sem} and RLD_{adv}) as the ratio between root length and soil volume, and root branching intensities (RBI_{sem} and RBI_{adv}) as the ratio between the number of root tips and the total root length.

We assessed heading date (Heading) and maturity date (Maturity) visually for each single-variety plot by recording the date at which spikes were visible for 50% of the plants and the date at which 50% of the peduncles were ripe, respectively. We converted these dates in growing degree days (GDD) by summing the daily average temperatures since sowing using a 0°C base temperature. Using a digital angle ruler positioned at the crown level, we measured aerial angle ($Angle_{aer}$) between the two most distant tillers, with two measurements per plot at heading. We measured root angle ($Angle_{root}$) at maturity: for each genotype, we extracted three plants from the soil and we measured the angle between the two most distant roots. Plant height (Height) was measured at maturity with three measurements per plot.

Leaf Area Index (LAI, m^2/m^2) representing the total leaf area per unit ground was measured by a frame-camera mounted on an unmanned aerial vehicle (UAV) that flew above the experimental field on 23 March 2018 (end of the tillering stage). After image analysis, we obtained one LAI value for each single-variety plot.

For all traits with replicated measurements, we obtained one value per genotype by averaging the replicates.

Correlations between yield components and STB severity

To assess how grain yield was affected by STB independently from genotype richness and allelic richness, we tested the relationship between yield components and their correlations with STB severity. To this end, we first computed grain yield, spike density, thousand kernel weight and STB severity in mixture plots by averaging the values of each component. We then used all pure stand and mixture data (226 complete observations) to compute crossed correlations between the four variables with Pearson correlation coefficients (Figure S8). Grain yield was strongly correlated with spike density ($r = 0.66$, $p < 0.001$), and to a lesser extent with thousand kernel weight ($r = 0.30$, $p < 0.001$). The correlation between STB severity and yield components was not significant. We then wanted to test if the effect of STB severity on grain yield would become significant when accounting for other sources of variations. We thus fitted a linear mixed model with grain yield as the response variable, STB severity, genotype richness, and their interaction as fixed effects, and genotypic pair identity as a random effect (Table S1). This random effect was structured according to a 226 x 226 matrix $\boldsymbol{K_p}$ (see "Genotyping data" section). Again, the effect of STB severity on grain yield was not significant (+6.04 g/m², s.e. = 12.06 g/m², p = 0.617), whereas genotype richness had a significant positive effect on grain yield (+ 86.21 g/m², s.e. = 38.98 g/m², p = 0.0288).

Checking for unwitting sampling effects

We checked whether the effect of allelic richness at cfn0881580 could originate from unwitting sampling effects resulting from either (i) the genotypes observed in bi-allelic mixtures being, by chance, the genotypes with the lowest yield and the highest STB severity among the 179 genotypes used in our experiment, (ii) the genotypes observed in mono-allelic mixtures being, by chance, the genotypes with the highest yield and lowest STB severity, or (iii) both (i) and (ii). To do so, we compared the subset of pure stands that were used in the three different types of mixtures, i.e., mono-allelic AA-AA, mono-allelic BB-BB, and bi-allelic AA-BB, with the whole set of pure stands to check whether they significantly differ in either GY or STB severity. We thus separated the dataset in 9 groups:

(1) all pure stands in which genotypes carried the "A" allele at cfn0881580 ("Mono AA")

(2) subset of pure stands in which genotypes carried the "A" allele and were used in mono-allelic mixtures ("Mono AA in Mix AA-AA")
(3) subset of pure stands in which genotypes carried the "A" allele and were used in bi-allelic mixtures ("Mono AA in Mix AA-BB")
(4) mixtures in which both genotypes carried the "A" allele ("Mix AA-AA")
(5) mixtures in which one genotype carried the "A" allele and the other genotype carried the "B" allele ("Mix AA-BB")
(6) mixtures in which both genotypes carried the "B" allele ("Mix BB-BB")
(7) subset of pure stands in which genotypes carried the "B" allele and were used in bi-allelic mixtures ("Mono BB in Mix AA-BB"),
(8) subset of pure stands in which genotypes carried the "B" allele and were used in mono-allelic mixtures ("Mono BB in Mix BB-BB")
(9) all pure stands in which genotypes carried the "B" allele ("Mono BB").

We compared the 9 groups for both grain yield and STB severity using a mixed model with the group as a fixed effect and the identity of the genotypic pair as a random effect (main text, Figure 4). The genotypic pair identity random effect was structured with a pairwise genetic similarity matrix $\boldsymbol{K_p}$ (see Material and Methods in the main manuscript). Post-hoc pairwise comparisons between the 9 groups were tested using the Tukey *p*-value adjustment (*glht()* function from the *multcomp* package).

Since groups (1), (2), (3), (7), (8), and (9) were not statistically different from each other for both grain yield and STB severity (main text, Figure 4), the allelic richness effect detected at cfn0881580 was not caused by a sampling effect.

REFERENCES

David J, Holtz Y, Ranwez V, Santoni S, Sarah G, Ardisson M, Poux G, Choulet F, Genthon C, Roumet P, *et al.* 2014. Genotyping by sequencing transcriptomes in an evolutionary pre-breeding durum wheat population. *Molecular Breeding* **34**: 1531–1548.

Ecarnot M, Compan F, Roumet P. **2013**. Assessing leaf nitrogen content and leaf mass per unit area of wheat in the field throughout plant cycle with a portable spectrometer. *Field Crops Research* **140**: 44–50.

Supplementary Figures and Tables

Table S1 | Effect of *Septoria tritici* blotch disease (STB) severity and genotype richness on grain yield. The model includes STB severity, genotype richness, and their interaction as fixed effects, and genotypic pair identity as a random effect. We here report parameter estimates along with their standard error (S.E.), *t*-values, *p*-values, and partial coefficients of determination (R^2p) for fixed effects, and estimated variances for random effects. Italic *p*-values are lower than 0.1 and higher than 0.05 whereas italic bold *p*-values are lower than 0.05.

	Grain yield (g/m²)				
**Fixed effects**	Estimate	S.E.	*t*-value	*p*-value	R^2_p
STB severity	6.04	12.06	0.501	0.617	0.001
Genotype richness	86.21	38.98	2.211	***0.028***	0.016
STB sev. x Genotype rich.	-53.29	27.60	-1.931	*0.055*	0.012

**Random effects**	Estimated Variance
Genotypic pair	4038
Residual	9359

Table S2 | Effects of focal and neighbour alleles at cfn0881580 on individual grain yield and *Septoria tritici* blotch disease (STB) severity in two-way mixtures of durum wheat. The model includes focal allele identity, neighbour allele identity, and the interaction between focal and neighbour allele identity as fixed effects, and focal genotype identity and neighbour genotype identity as random effects. We here report parameter estimates (Est.) along with their standard error (S.E.), *t*-values (*t*-val.), *p*-values (*p*-val.), and partial coefficients of determination (R^2_p) for fixed effects, and estimated variances for random effects. Italic *p*-values are lower than 0.1 and higher than 0.05 whereas italic bold *p*-values are lower than 0.05.

	Grain yield (g/m²)					STB severity				
**Fixed effects**	Est.	S.E.	*t*-val.	*p*-val.	R_p^2	Est.	S.E.	*t*-val.	*p*-val.	R_p^2
Focal allele	-14.18	9.81	-1.445	0.149	0.006	0.28	0.08	3.562	***<0.001***	0.103
Neighbour allele	-26.92	8.83	-3.047	***0.002***	0.023	0.26	0.08	3.353	***0.001***	0.088
Foc. allele x Nei. allele	22.54	5.91	3.811	***<0.001***	0.031	-0.24	0.05	-4.437	***<0.001***	0.137

**Random effects**	Estimated Variance	Estimated Variance
Focal genotype	2094.1	0.030
Neighbour genotype	300.1	0.011
Residual	10544.0	0.270

Table S3 | Effects of neighbour allele at cfn0881580 on focal grain yield and *Septoria tritici* blotch disease (STB) severity in two-way mixtures of durum wheat. The analysis was performed separately for focal genotypes bearing the "AA" or "BB" alleles, respectively. The model includes neighbour allele identity as a fixed effect, and focal genotype identity and neighbour genotypes identity as random effects. We here report parameter estimates (Est.) along with their standard error (S.E.), *t*-values (*t*-val.), *p*-values (*p*-val.), and partial coefficients of determination (R^2_p) for fixed effects, and estimated variances for random effects. Italic *p*-values are lower than 0.1 and higher than 0.05 whereas italic bold *p*-values are lower than 0.05.

	**Grain yield (g/m²)**					**STB severity**				
**FOCAL = AA**										
**Fixed effects**	Est.	S.E.	*t*-val.	*p*-val.	R_p^2	Est.	S.E.	*t*-val.	*p*-val.	R_p^2
Neighbour allele	-54.00	17.20	-3.140	***0.002***	0.051	0.52	0.15	3.332	***0.002***	0.174
**Random effects**	Estimated Variance					Estimated Variance				
Focal genotype	1062.4					0.019				
Neighbour genotype	0.0					0.028				
Residual	12009.7					0.230				
**FOCAL = BB**										
**Fixed effects**	Est.	S.E.	*t*-val.	*p*-val.	R_p^2	Est.	S.E.	*t*-val.	*p*-val.	R_p^2
Neighbour allele	37.38	16.63	2.247	***0.026***	0.082	-0.48	0.15	-3.190	***0.002***	0.146
**Random effects**	Estimated Variance					Estimated Variance				
Focal genotype	2924.7					0.051				
Neighbour genotype	440.1					0.091				
Residual	9268.4					0.066				

Table S4 | Effects of allelic richness at cfn0881580 and genotype richness on grain yield and *Septoria tritici* blotch disease (STB) severity. The mixed model includes allelic richness at cfn0881580 and genotype richness as fixed effects, and genotypic pair identity as a random effect. We here report parameter estimates (Est.) along with their standard error (S.E.), *t*-values (*t*-val.), *p*-values (*p*-val.), and partial coefficients of determination (R^2_p) for fixed effects, and estimated variances for random effects. Italic *p*-values are lower than 0.1 and higher than 0.05 whereas italic bold *p*-values are lower than 0.05.

	Grain yield (g/m²)					STB severity				
**Fixed effects**	Est.	S.E.	*t*-val.	*p*-val.	R_p^2	Est.	S.E.	*t*-val.	*p*-val.	R_p^2
Allelic richness	-52.30	12.16	-4.300	***<0.001***	0.023	0.43	0.14	3.070	***0.002***	0.014
Genotype richness	37.01	10.71	3.456	***<0.001***	0.014	-0.67	0.11	-6.141	***<0.001***	0.055

**Random effects**	Estimated Variance	Estimated Variance
Genotypic pair	9339	0.6101
Residual	6885	0.2700

Table S5 | Effects of allelic richness at cfn0881580 and genotype richness on spike density and thousand kernel weight. The mixed model includes allelic richness at cfn0881580 and genotype richness as fixed effects and genotypic pair identity as a random effect. We here report parameter estimates (Est.) along with their standard error (S.E.), *t*-values (*t*-val.), *p*-values (*p*-val.), and partial coefficients of determination (R^2_p) for fixed effects, and estimated variances for random effects. Italic *p*-values are lower than 0.1 and higher than 0.05 whereas italic bold *p*-values are lower than 0.05.

	Spike density (nb of spikes/m²)					Thousand kernel weight (g)				
**Fixed effects**	Est.	S.E.	*t*-val.	*p*-val.	R_p^2	Est.	S.E.	*t*-val.	*p*-val.	R_p^2
Allelic richness	-21.21	6.01	-3.528	***<0.001***	0.010	-0.42	0.35	-1.223	0.222	0.000
Genotype richness	18.95	5.26	3.602	***<0.001***	0.010	0.35	0.30	1.185	0.237	0.000
**Random effects**	Estimated Variance					Estimated Variance				
Genotypic pair	4637					78.578				
Residual	1611					4.745				

Table S6 | Association between phenotypic variation in single-variety plots and allelic variation at cfn0881580. 20 traits were tested including 7 aboveground traits, 11 root traits, and 2 phenological traits. Estimates (Est.), standard error (S.E.), *t*-values (*t*-val.), and *p*-values (*p*-val.) refer to the effect of allelic variation at cfn0881580 on the tested trait. *p*-values were computed with the Benjamini-Hochberg correction to account for multiple testing.

	**Trait**	**Unit**	Est.	S.E.	*t*-val.	*p*-val.
Aboveground	Ear. bio.	$g.ind^{-1}$	-0.03	0.03	-1.199	0.5791
	Till. nb.	nb $till.ind^{-1}$	-0.13	0.07	-1.906	0.3889
	$Angle_{aer}$	°	-0.42	0.22	-1.933	0.3889
	SLA	$m^2.kg^{-1}$	0.07	0.25	0.271	0.8501
	LNC	%	0.02	0.03	0.820	0.6433
	Height	cm	0.12	0.31	0.393	0.8501
	LAI	$m^{-2}.m^{-2}$	0.01	0.01	1.129	0.5791
Belowground	RLD_{sem}	$cm.cm^{-3}$	0.01	0.01	1.239	0.5791
	RLD_{adv}	$cm.cm^{-3}$	0.00	0.01	-0.293	0.8501
	SRL_{sem}	$m.g^{-1}$	1.32	1.87	0.705	0.6884
	SRL_{adv}	$m.g^{-1}$	1.55	0.62	2.501	0.2662
	RTD_{sem}	$g.cm^{-3}$	0.00	0.00	-0.005	0.9956
	RTD_{adv}	$g.cm^{-3}$	0.00	0.00	-1.482	0.5602
	RBI_{sem}	$tips.cm^{-1}$	0.00	0.01	0.244	0.8501
	RBI_{adv}	$tips.cm^{-1}$	0.00	0.01	-0.471	0.8501
	$Diam_{sem}$	mm	0.00	0.00	-0.857	0.6433
	$Diam_{adv}$	mm	-0.01	0.00	-1.493	0.5602
	$Angle_{root}$	°	-1.10	1.36	-0.812	0.6433
Phenology	Heading	GDD	-4.89	3.54	-1.381	0.5630
	Maturity	GDD	3.26	3.39	0.963	0.6433

Table S7 | Monthly meteorological data measured at Mauguio (FRANCE, 43°36’ N, 3°59’ E): solar radiation (Rad.), maximum temperature (Tmax), minimum temperature (Tmin), mean temperature (Tmean), and precipitations (Prec). Data were extracted from the INRA CLIMATIK database (*https://intranet.inra.fr/climatik_v2)*.

	**Nov.**	**Dec.**	**Jan.**	**Feb.**	**Mar.**	**Apr.**	**May**	**Jun.**
Rad (dJ.cm^{-2})	2409	1681	1735	2617	4096	5056	6348	7783
Tmax (°C)	15.7	12.2	14.5	10	15.2	19.7	22.8	27.5
Tmin (°C)	5.7	1.3	7.1	1.3	6.4	9.5	12.9	15.9
Tmean (°C)	10.3	6.3	10.8	5.2	10.6	14.7	17.5	21.9
Prec (mm)	30	12	202	86.5	103	82.5	65.5	51.5

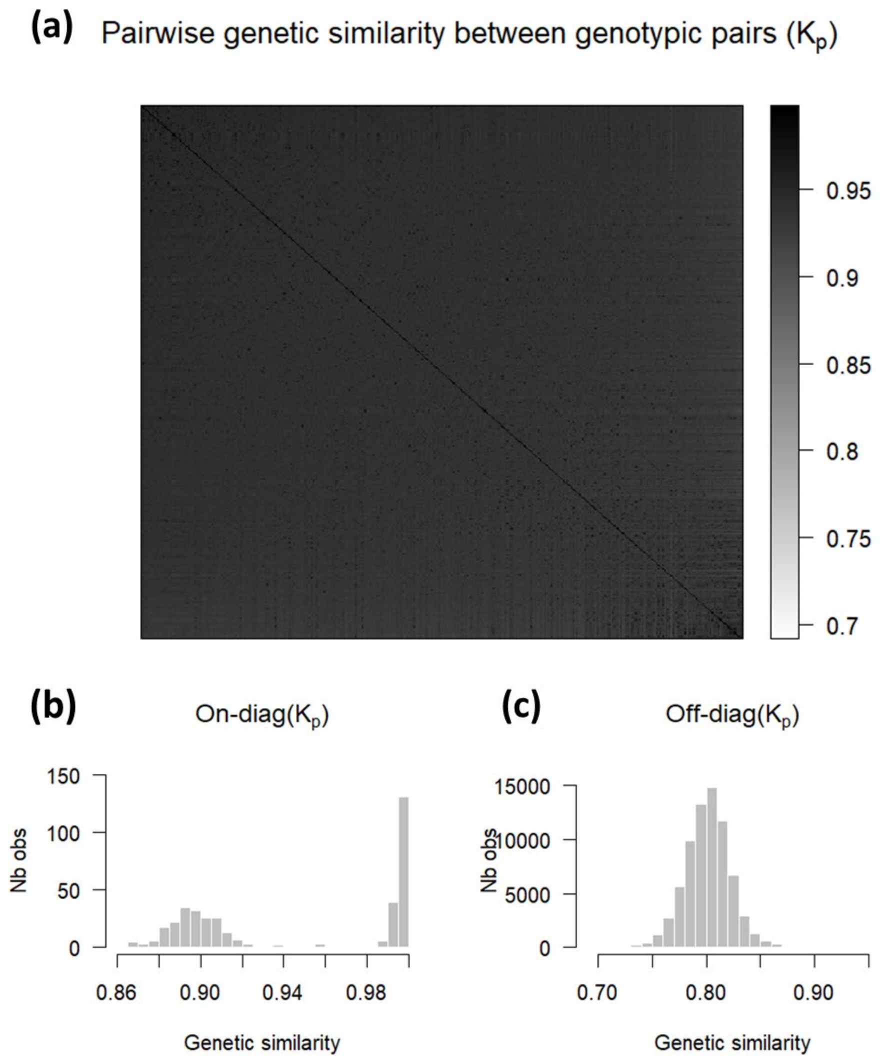

Figure S1 | Representation of the pairwise genetic similarity between genotypic pairs. (a) heatmap of the ordered 381 x 381 K_p matrix where $K_p[i,j]$ represents the genetic similarity between genotypic pairs i and j. Values increase from white to black. **(b)** Distribution of the "on-diagonal" values of K_p. **(c)** Distribution of the "off-diagonal" values of K_p.

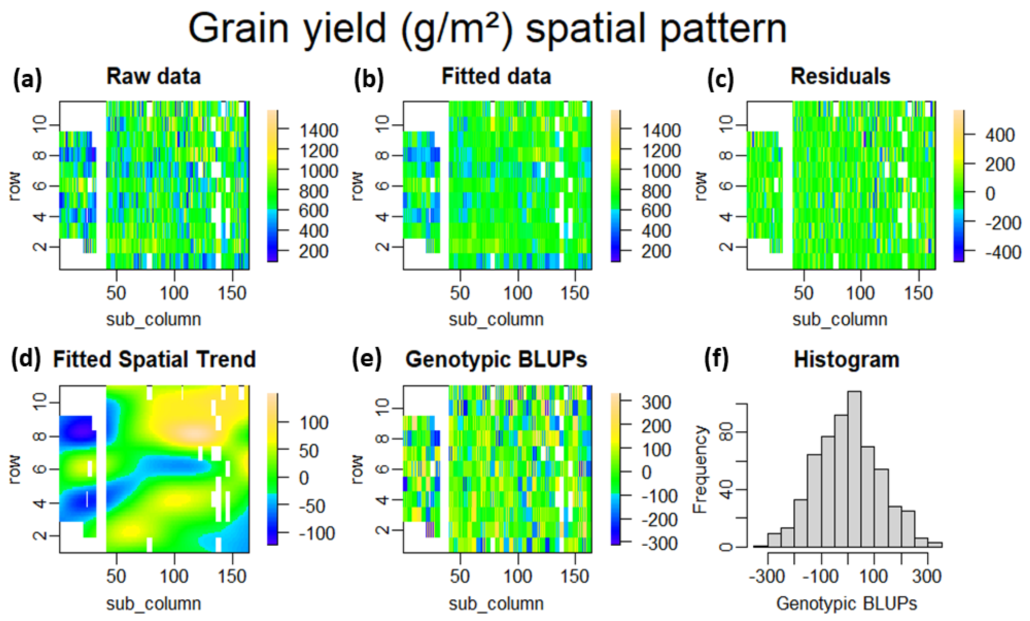

Figure S2 | Representation of the field spatial effect modelled by *SpATS* for grain yield. Each plot is represented by its 2-D coordinates (row and sub-column, see the description of the design in the main text), **(a)** raw grain yield data, **(b)** grain yield fitted with the model, **(c)** residuals from the model, **(d)** smooth bivariate surface estimated by the model, **(e)** genotypic BLUPs extracted from the model, **(f)** Distribution of the genotypic BLUPs. Blanks correspond to plots removed from the dataset due to sowing or sampling problems.

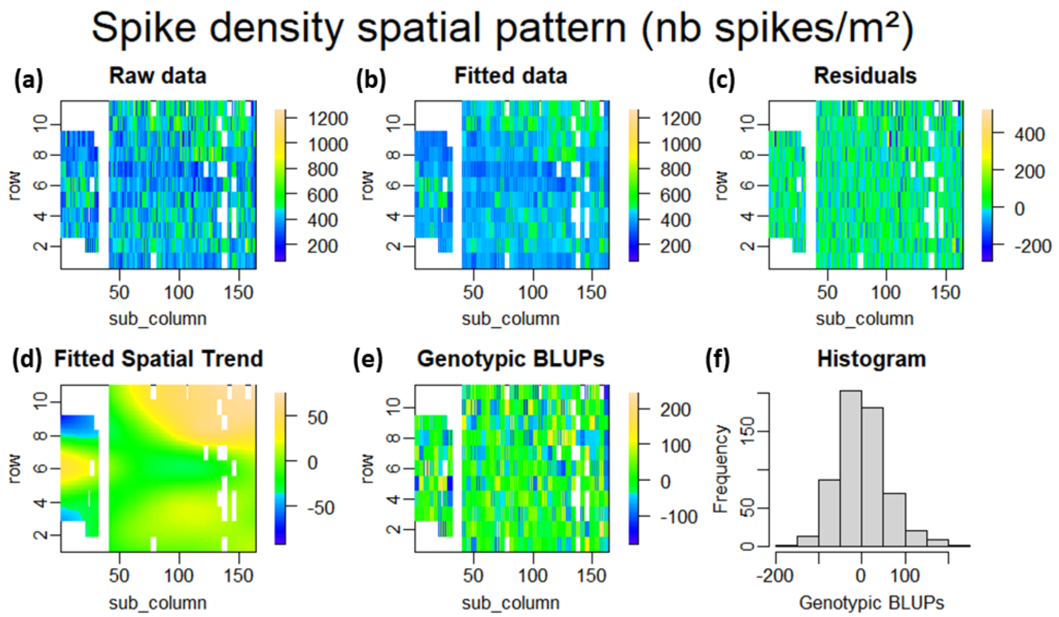

Figure S3 | Representation of the field spatial effect modelled by *SpATS* for spike density. Each plot is represented by its 2-D coordinates (row and sub-column, see the description of the design in the main text), **(a)** raw spike density data, **(b)** spike density fitted with the model, **(c)** residuals from the model, **(d)** smooth bivariate surface estimated by the model, **(e)** genotypic BLUPs extracted from the model, **(f)** Distribution of the genotypic BLUPs. Blanks correspond to plots removed from the dataset due to sowing or sampling problems.

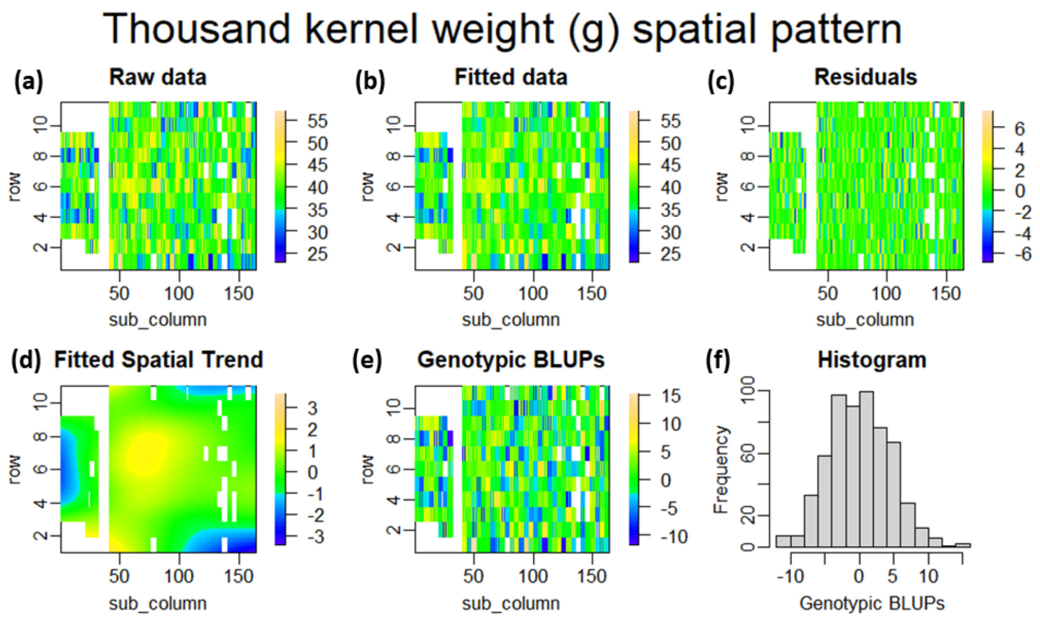

Figure S4 | Representation of the field spatial effect modelled by *SpATS* for thousand kernel weight. Each plot is represented by its 2-D coordinates (row and sub-column, see the description of the design in the main text), **(a)** raw thousand kernel weight data, **(b)** thousand kernel weight fitted with the model, **(c)** residuals from the model, **(d)** smooth bivariate surface estimated by the model, **(e)** genotypic BLUPs extracted from the model, **(f)** Distribution of the genotypic BLUPs. Blanks correspond to plots removed from the dataset due to sowing or sampling problems.

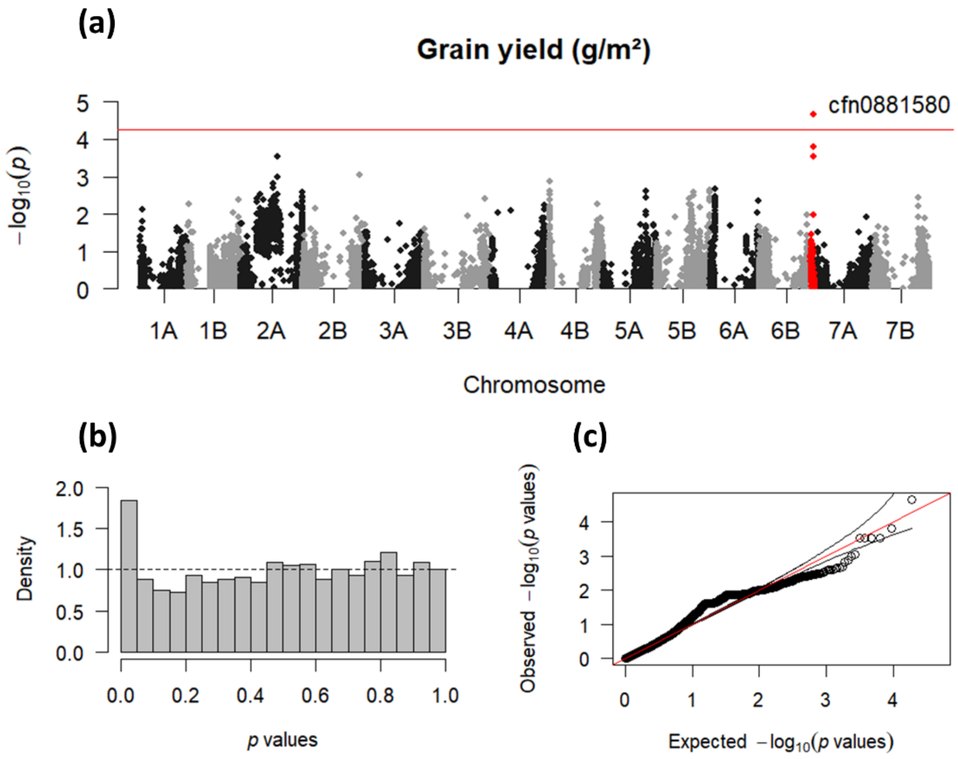

Figure S5 | Locus-by-locus analysis of allelic richness effect on grain yield. **(a)** Manhattan plot reporting *p*-values (-log10 transformed) for the association tests between grain yield and allelic richness at 18,868 SNPs distributed along the durum wheat genome. The solid red line represents the Family-Wise Error Rate (FWER) of 5% computed with the Galwey method. The points highlighted in red are the most significant SNP (cfn0881580) and the surrounding SNPs at ± 40 Mb. **(b)** Distribution of the 18,868 *p*-values obtained with the genome-wide association test. The dotted line represents the theoretical uniform *p*-value distribution under H_0 (all SNPs effects are null). **(c)** Q-Q plot representing the observed vs expected quantiles of the *p*-value distribution. Solid lines show the expected quantiles under the null hypothesis (red) and their 95% confidence interval (black).

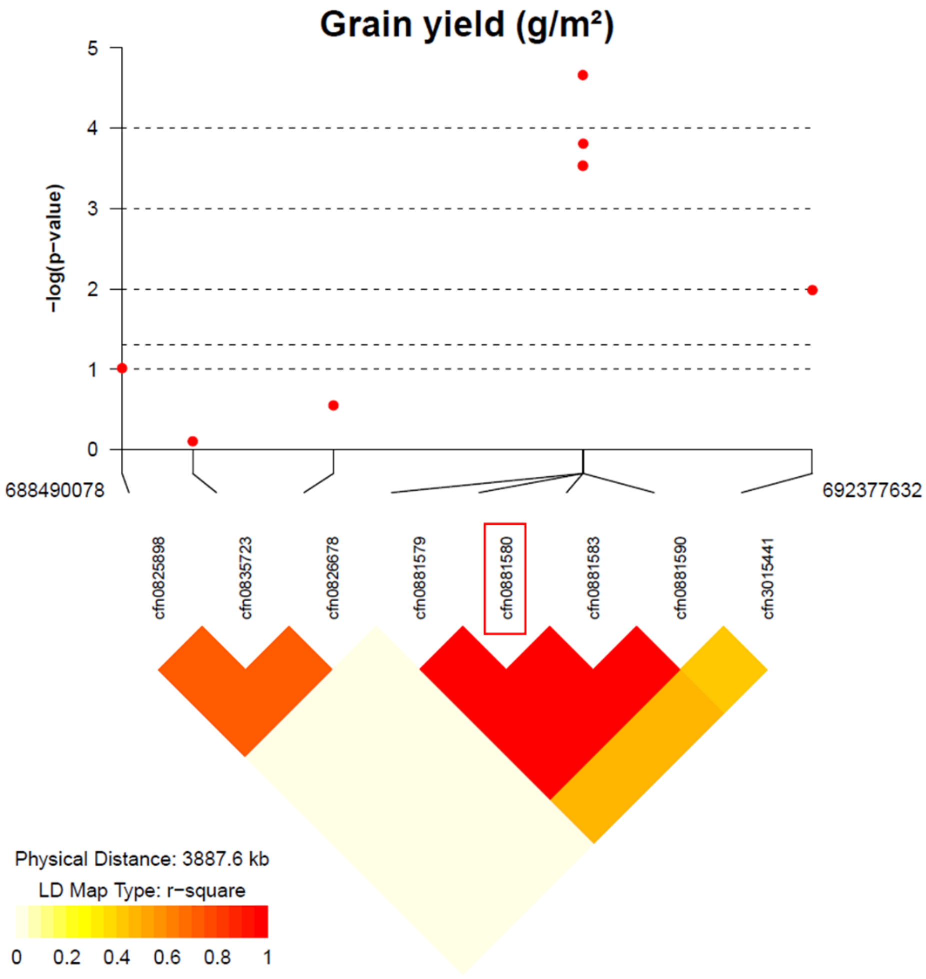

Figure S6 | Linkage disequilibrium (LD) in the genomic region where allelic richness is significantly associated with grain yield. The upper part of the chart represents the *p*-values (-log10 transformed) of the association test between allelic richness and grain yield for the 7 SNPs present in the region. The bottom part depicts the LD (r^2) between the different SNPs, with white squares representing low LD and red squares representing high LD. SNPs positions reflect their relative physical position on the chromosome. The SNP with the most significant signal, cfn0881580, is framed in red.

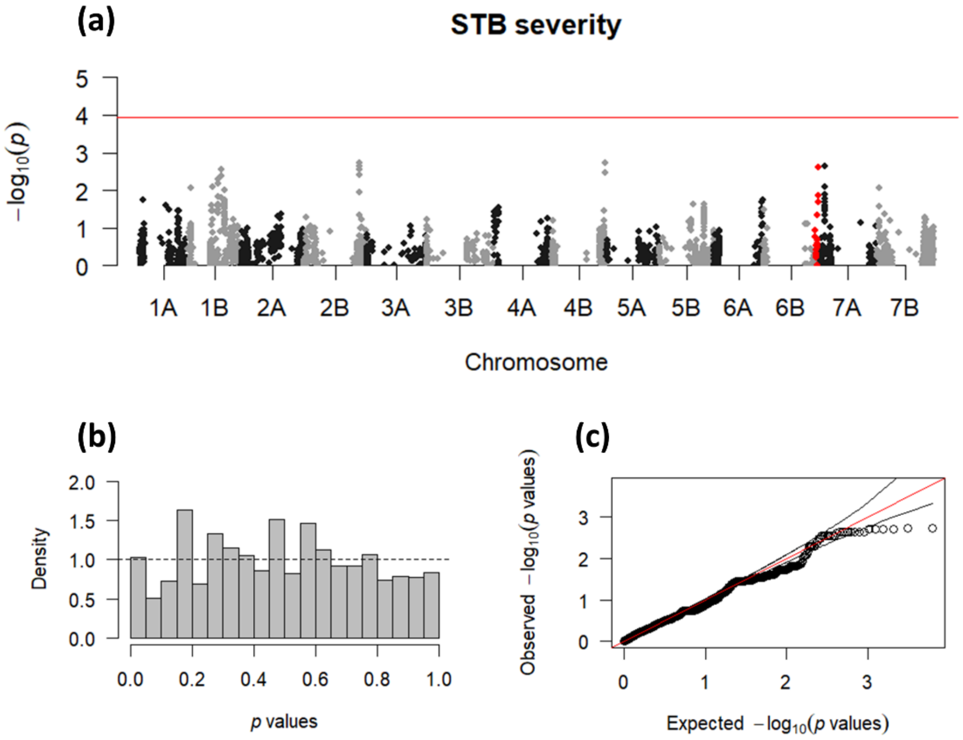

Figure S7 | Locus-by-locus analysis of allelic richness effect on Septoria tritici blotch (STB) severity. (a) Manhattan plot reporting *p*-values (-log10 transformed) for the association tests between STB severity and allelic richness at 6,193 SNPs distributed along the durum wheat genome (Note that we could not use the 18,868 SNPs for STB severity since this variable was only available for 226 plots). The solid red line represents the Family-Wise Error Rate (FWER) of 5% computed with the Galwey method. The SNP significantly associated with grain yield (cfn0881580) and all surrounding SNPs at ± 40 Mb are highlighted in red. **(b)** Distribution of the 6,193 *p*-values obtained with the genome-wide association test. The dotted line represents the theoretical uniform *p*-value distribution under H_0 (all SNPs effects are null). **(c)** Q-Q plot representing the observed vs expected quantiles of the *p*-value distribution. Solid lines show the expected quantiles under the null hypothesis (red) and their 95% confidence interval (black).

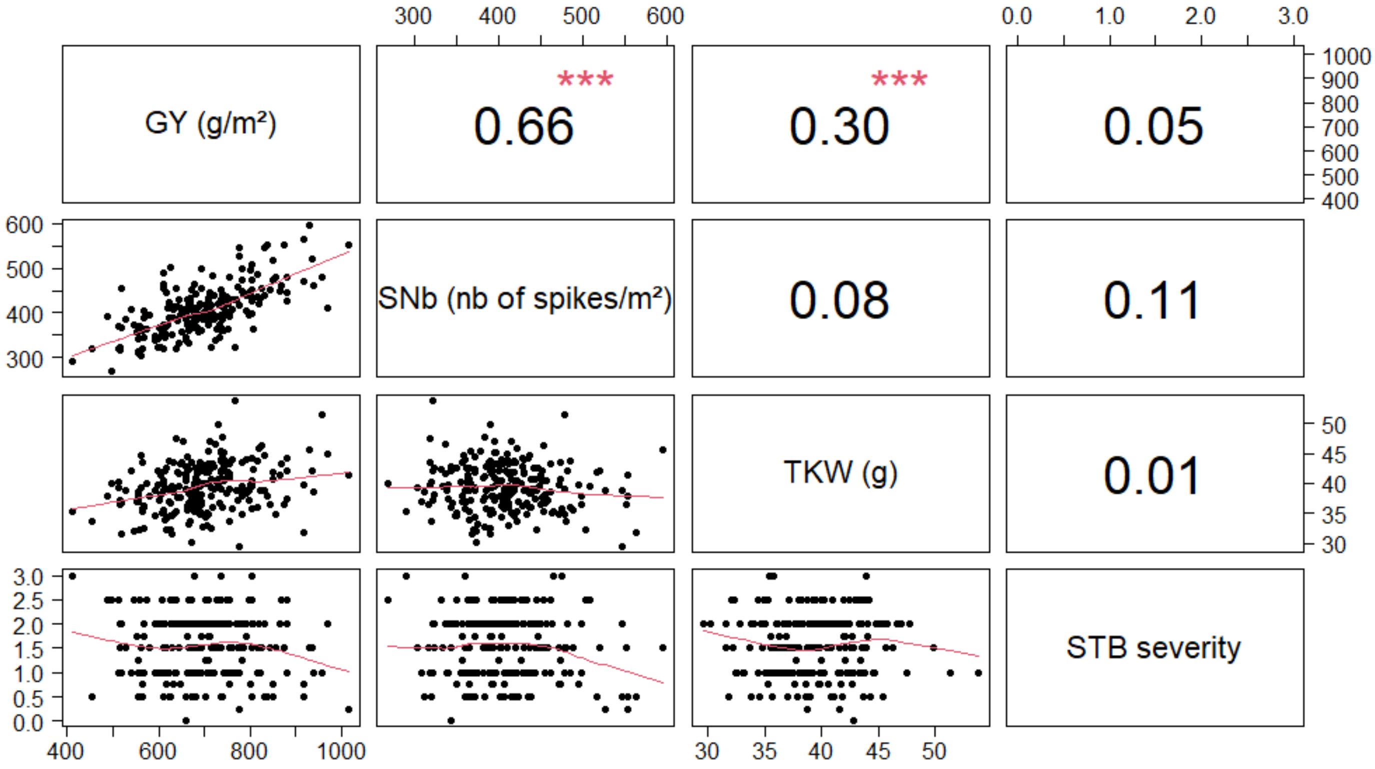

Figure S8 | Pairwise correlations between grain yield (GY, g/m²), spike density (SNb, nb spikes/m²), thousand kernel weight (TKW, g), and Septoria tritici blotch disease (STB) severity. Pearson correlation coefficients are reported in the upper triangle (*** $p \leq 0.001$). The lower triangle represents pairwise scatter plots with smooth curves in red. We here used data from 166 pure stands and 60 mixtures ($n = 226$).

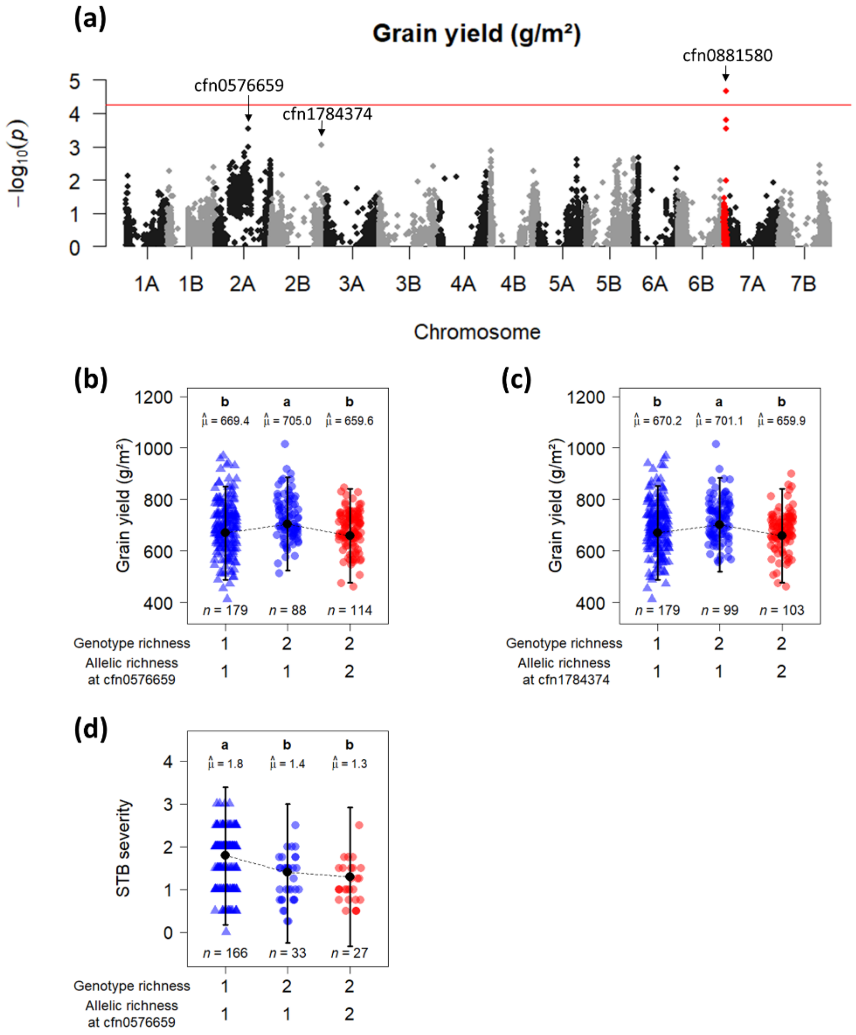

Figure S9 | Effect of allelic richness at the two most significant SNPs after cfn0881580. (a) Manhattan plot reporting *p*-values (-log10 transformed) for the association tests between grain yield and allelic richness at 18,868 SNPs distributed along the durum wheat genome. The solid red line represents the Family-Wise Error Rate (FWER) of 5% computed with the Galwey method. The most significant SNPs, cfn088580, is highlighted in red. The next two most significant SNPs are annotated: cfn0576659 on chr. 2A and cfn1784374 on chr. 2B. **(b)** and **(c)** Grain yield over the different combinations of allelic richness at cfn0576659 **(b)** and cfn1784374 **(c)** and genotype richness. **(d)** STB severity over the different combinations of allelic richness at cfn0576659 and genotype richness. We could not test the effect of allelic richness at cfn1784374 on STB severity because this SNP had very unbalanced allelic representation in the STB dataset. Genotype richness is quantified as the number of genotypes in the plot, while allelic richness is quantified as the number of alleles at in the plot. Point shapes: triangles = pure stand plots, circles = mixture plots. Point colors: blue = mono-allelic plots, red = bi-allelic plots. Black points and error bars represent the estimated marginal means and their 95% confidence interval. *n*: number of observations in each category, $\hat{\mu}$: marginal means. Categories with different letters are significantly different at $p < 0.05$ (Tukey adjustment).
